# Risk of longer-term neurological conditions in the Deepwater Horizon Oil Spill Coast Guard Cohort Study – Five years of follow-up

**DOI:** 10.1186/s12940-022-00941-0

**Published:** 2023-01-25

**Authors:** Hristina Denic-Roberts, Lawrence S. Engel, Jeanine M. Buchanich, Rachel G. Miller, Evelyn O. Talbott, Dana L. Thomas, Glen A. Cook, Tina Costacou, Jennifer A. Rusiecki

**Affiliations:** 1 Department of Epidemiology, School of Public Health, University of Pittsburgh, Pittsburgh, Pennsylvania USA; 2 Department of Preventive Medicine and Biostatistics, Uniformed Services University of the Health Sciences, 4301 Jones Bridge Road, Room E-2009, Bethesda, MD 20814 USA; 3 Department of Epidemiology, Gillings School of Public Health, University of North Carolina, Chapel Hill, North Carolina USA; 4 Department of Biostatistics, School of Public Health, University of Pittsburgh, Pittsburgh, Pennsylvania USA; 5 United States Coast Guard Headquarters, Directorate of Health, Safety, and Work Life, Washington, D.C., USA; 6 Department of Neurology, Uniformed Services University of the Health Sciences, Bethesda, Maryland USA

**Keywords:** Deepwater Horizon Oil Spill, Crude Oil, Dispersants, U.S. Coast Guard, Spill Responders, Neurological Conditions

## Abstract

**Background:**

Long-term neurological health risks associated with oil spill cleanup exposures are largely unknown. We aimed to investigate risks of longer-term neurological conditions among U.S. Coast Guard (USCG) responders to the 2010 *Deepwater Horizon* (DWH) oil spill.

**Methods:**

We used data from active duty members of the DWH Oil Spill Coast Guard Cohort Study (*N*=45224). Self-reported oil spill exposures were ascertained from post-deployment surveys. Incident neurological outcomes were classified using International Classification of Diseases, 9th Revision, codes from military health encounter records up to 5.5 years post-DWH. We used Cox Proportional Hazards regression to calculate adjusted hazard ratios (aHR) and 95% confidence intervals (CI) for various incident neurological diagnoses (2010–2015). Oil spill responder (*n*=5964) vs. non-responder (*n*= 39260) comparisons were adjusted for age, sex, and race, while within-responder comparisons were additionally adjusted for smoking.

**Results:**

Compared to those not responding to the spill, spill responders had reduced risks for *headache* (aHR=0.84, 95% CI: 0.74-0.96), *syncope and collapse* (aHR=0.74, 95% CI: 0.56-0.97), and *disturbance of skin sensation* (aHR=0.81, 95% CI: 0.68-0.96). Responders reporting ever (*n*=1068) vs. never (*n*=2424) crude oil inhalation exposure were at increased risk for several individual and grouped outcomes related to headaches and migraines (aHR range: 1.39-1.83). Crude oil inhalation exposure was also associated with elevated risks for an inflammatory nerve condition, *mononeuritis of upper limb and mononeuritis multiplex* (aHR=1.71, 95% CI: 1.04-2.83), and *tinnitus* (aHR=1.91, 95% CI: 1.23-2.96), a condition defined by ringing in one or both ears. Risk estimates for those neurological conditions were higher in magnitude among responders reporting exposure to both crude oil and oil dispersants than among those reporting crude oil only.

**Conclusion:**

In this large study of active duty USCG responders to the DWH disaster, self-reported spill cleanup exposures were associated with elevated risks for longer-term neurological conditions.

**Supplementary Information:**

The online version contains supplementary material available at 10.1186/s12940-022-00941-0.

## Background

On April 20^th^, 2010, the semi-submersible offshore oil drilling rig *Deepwater Horizon* (DWH) experienced an explosion near the coast of Louisiana that killed 11 workers and injured 15 others [1, 2]. This disaster caused the largest marine oil spill in U.S. history. After 87 days of fresh crude oil flowing into the Gulf of Mexico, the well was effectively capped on July 15^th^, 2010 [1]. It has been estimated that a total of 185 to 210 million gallons, or approximately 4.4 to 5 million barrels of crude oil, were discharged into the Gulf over the course of the spill [1–5]. In addition, approximately 1.8 million gallons of chemical oil dispersants COREXIT 9500 and 9527A were applied on the water surface and subsea, in an effort to rapidly disperse the spilled oil [1]. Many federal agencies were involved in the spill cleanup efforts, led by the U.S. Coast Guard (USCG), which deployed nearly 9000 of its own service members [1]. The USCG responders, along with tens of thousands of other cleanup workers and volunteers, as well as Gulf coast residents, were potentially exposed to various harmful chemicals, including crude oil and chemical dispersants, which may have adversely affected their health.

Crude oil is a complex mixture of thousands of chemical compounds including volatile organic compounds (VOCs), polycyclic aromatic hydrocarbons (PAHs), hydrogen sulfide, and heavy metals [6]. Several crude oil constituents, including VOCs, hydrogen sulfide, and heavy metals, have been found to affect both the central nervous system (CNS) and the peripheral nervous system (PNS). For instance, exposure to the VOC chemicals benzene, toluene, ethylbenzene, and xylenes (commonly referred to as BTEX) has been linked to adverse neurological effects, such as headaches, dizziness, cognitive impairment, and vision and hearing damage [7–13]. Hydrogen sulfide exposure has been associated with acute and chronic CNS symptoms including headaches, poor memory, poor attention span, and poor motor function [14]. Heavy metals present in crude oil, including copper and lead, have also been linked to adverse CNS and PNS effects [15, 16]. Additionally, repeated or excessive exposure to 2-butoxyethanol, a constituent of oil dispersant COREXIT 9527A, has been associated with CNS depression and headaches [17, 18]. Because of the neurotoxic properties of crude oil and dispersant constituents, understanding the relationship between oil spill exposures and neurological health is of crucial importance for preventing neurological damage among people who will be involved in cleanup of future oil spills and those residing in the proximity of future oil spill disasters.

Even though oil spills continue to occur worldwide and to disturb environmentally sensitive areas that are already threated by climate change, the adverse health effects among exposed response workers, volunteers, and affected communities, in particular long-term health effects, are largely unknown. The majority of studies that assessed health effects among those involved in oil spill disasters have been cross-sectional and have largely focused on *acute* physical and mental health symptoms [19, 20]. While most frequently reported acute physical symptoms among oil spill-exposed workers/volunteers and affected residents have been related to the respiratory system [21–41], several cross-sectional studies have also reported acute neurological symptoms such as headaches, migraines, nausea, dizziness, difficulty concentrating, blurred/impaired vision, memory loss, confusion, and numbness and tingling [21, 22, 24–26, 28, 30–34, 36–40, 42, 43]. Headaches were the most commonly reported acute neurological symptom across studies, however, many also observed elevated prevalence of symptoms consistent with both CNS (e.g., dizziness, nausea) and PNS impairments (e.g., numbness/tingling, impaired walking).

While studies of acute neurological symptoms have consistently observed positive associations with various measures of oil spill exposure (e.g., duration of cleanup, crude oil exposure), to our knowledge, only two studies to date, both conducted after the DWH oil spill, have evaluated longer-term neurological outcomes two to six years following participation in oil spill cleanup [40, 44]. In the Deepwater Horizon Oil Spill Coast Guard (DWH-CG) Cohort Study, Rusiecki et al. investigated associations between oil spill exposures and incident neurological conditions up to two years post-DWH using objective military health encounter records [40]. Compared to active duty USCG responders who reported no exposure to crude oil via any route, the crude oil-exposed responders had a non-significantly elevated risk of headaches and migraines (adjusted risk ratio=1.35, 95% CI: 0.95-1.92). In a recent investigation of the Gulf Long Term Follow-up (GuLF) Study, Quist and colleagues [44] assessed neurobehavioral performance at a clinical exam four to six years after the DWH spill response and cleanup. Overall, the GuLF Study investigators found that cleanup jobs with higher oil spill exposures, as well as higher estimated total hydrocarbon exposure levels, were modestly associated with impaired neurobehavioral function including impairments in attention and memory, executive function, working memory, effort-related motivation, and response speed/coordination [44].

Because longer-term neurological health effects associated with exposure to oil spill chemicals, such as crude oil and dispersants, are understudied and largely unknown, but biologically plausible, the aim of our study was to further investigate these associations prospectively among USCG responders to the DWH oil spill in a well-established cohort study, the DWH-CG Cohort Study [40]. Specifically, we aimed to assess risks of longer-term neurological conditions in relation to the spill response and self-reported exposures to crude oil and dispersants, using post-deployment survey exposure data and objective military health encounter data up to five and a half years post-spill. Although our previous study examined associations between oil spill exposures (i.e., the oil spill response and exposure to crude oil via any route) and incident neurological conditions in the DWH-CG Cohort, that investigation was limited to two years of follow-up and two broad categories of neurological outcomes based on military health records: headaches and migraines, and neurological conditions excluding headaches/migraines [40]. In the present study, we expand upon our previous investigation by including longer follow-up (5.5 years), a range of neurological diseases and symptoms, and more specific exposure metrics, including crude oil inhalation and combined exposure to crude oil and dispersants.

## Methods

### Study population and study design

The DWH-CG Cohort has been described in detail elsewhere [40]. Briefly, this cohort was established with an aim of examining associations between the DWH oil spill response and both acute health symptoms and longer-term health conditions. The DWH-CG Cohort consists of 8696 USCG members who responded to the DWH oil spill for at least one day (i.e., responders) and 44823 USCG personnel who were either on active duty or in the Selected Reserve for at least one day during the main operational phase of the cleanup (April 20, 2010 – December 17, 2010 [1]), but who did not deploy to the spill (i.e., non-responders). For the current prospective study, we were able to include only active duty responders (*n*=5964, 68.6%) and non-responders (*n*=39260, 87.6%) because only active duty military personnel, and not the Select Reservists, have comprehensive medical coverage through the Military Health System (MHS) and, thus, ongoing health encounter data available for querying neurological diagnoses. Detailed information about the benefits of MHS, a healthcare system designed for equal access, was previously published [45].

This study was approved by the Institutional Review Boards (IRB) of the Uniformed Services University (USU), the U.S. Coast Guard, and the University of North Carolina, Chapel Hill. A waiver for informed consent was approved by the USU IRB.

### Exposure assessment

Our first exposure metric included the spill response work comparing all active duty responders (*n*=5964) to non-responders (*n*=39260). The rest of the oil spill related exposures were applicable to responders only. For the within-responder comparisons, we relied on self-reported exposures to crude oil/oily water (henceforth referred to as “crude oil”) and oil dispersants (henceforth referred to as “dispersants”) that were ascertained from two, previously described [40], post-deployment surveys.

Briefly, Survey 1 was launched on June 25, 2010 and Survey 2 on November 1, 2010. The questions on the two surveys were similar, however, Survey 1 assessed self-reported exposures to crude oil via different routes (i.e., inhalation, direct skin contact, ingestion, and submersion) on a binary scale (never, ever), while Survey 2 evaluated these crude oil exposures on a 5-point Likert scale (never, rarely, sometimes, most of the time, and all of the time). Self-reported exposure of coming into contact with dispersants, also on a 5-point Likert scale, was ascertained only on Survey 2. There were 3492 active duty responders who completed at least one of the two surveys, however, 390 active duty responders completed only Survey 1, and therefore had no information on dispersants.

For the within-responder analyses, we used several survey-based exposure metrics: 1) crude oil exposure via any route (i.e., inhalation, direct skin contact, ingestion, or submersion), 2) crude oil exposure via inhalation, 3) crude oil exposure via direct skin contact, 4) crude oil exposure via submersion, and 5) combined crude oil/dispersants exposure. For the crude oil exposure via any route and via submersion (*n*=3492), we combined self-reported data from both post-deployment surveys and created the following exposure metrics: ever (combining "ever" from Survey 1 and “rarely,” "sometimes," "most of the time,” or "all of the time" from Survey 2) vs. never (combining "never" from Survey 1 and "never" from Survey 2). For analyses relating to crude oil exposure via inhalation and via direct skin contact (*n*=3492), we combined self-reported data from both surveys and created the following exposure metrics: ever (combining "ever" from Survey 1 and "sometimes," "most of the time,” or "all of the time" from Survey 2) vs. never (combining "never" from Survey 1 and "never" or "rarely" from Survey 2). We combined the responses of “rarely” and “never” for the crude oil exposures via inhalation and via direct skin contact metrics because these two exposures were the most commonly reported.

For the combined crude oil/dispersants exposure metric, we created the following exposure groups: “Neither” (i.e., reported “never” exposure to crude oil via any route *and* “never” exposure to dispersants); “Oil only” (i.e., reported “ever” exposure to crude oil via any route *and* “never” exposure to dispersants); and “Both” (i.e., reported “ever” exposure to crude oil via any route *and* exposure to dispersants of “rarely”, “sometimes,” “most of the time,” or “all of the time”). For this comparison, the “Neither” group was the referent category. Because there were only 20 responders who reported any exposure to dispersants (i.e., “rarely” or greater) but no exposure to crude oil (i.e., “never” exposure to crude oil via any route), we did not create a “Dispersants only” exposure category.

### Outcome assessment

We ascertained neurological outcomes from the Military Health System Data Repository (MDR), a medical health encounter data repository maintained by the military [40, 45, 46]. The MDR contains data from inpatient and outpatient health encounters obtained in both military treatment facilities and clinics (“direct care”) and in civilian treatment facilities for which care is billed to the military (“purchased care”). For all active duty cohort members, we obtained full medical health encounter coverage for a period between October 1, 2007 (i.e., ~ two and a half years before the DWH spill) and September 30, 2015 (i.e., five and a half years post-spill) by combining MDR data from four major sources: 1) inpatient direct/military care, 2) outpatient direct/military care, 3) inpatient purchased/civilian care, and 4) outpatient purchased/civilian care. For the time period we queried, health encounter MDR diagnoses were coded using the International Classification of Diseases, 9^th^ Revision (ICD-9) codes. We focused on chronic neurological diseases and symptoms classified by three-, four-, or five-digit ICD-9 codes. We considered individual and grouped ICD-9 codes for various neurological conditions including migraines and headaches, conditions impairing memory, signs and symptoms involving cognition, peripheral nerve disorders, visual, hearing, and disturbance of skin sensation, as well as conditions affecting balance/gait. A full listing of individual and grouped diseases and symptoms that we evaluated, along with the corresponding ICD-9 codes, is provided in Supplemental Table 1.

The incident case definition for classifying neurological outcomes included at least one inpatient encounter or two outpatient encounters for a specific individual neurological disease/symptom or a group of neurological diseases/symptoms. We only retained outcomes with at least 10 cases among all responders and 10 cases among all non-responders, in order to avoid data sparsity issues. Prevalent cases among the responders and non-responders who had a pre-existing neurological condition documented in MDR before the spill (October 1, 2007 - April 20, 2010), ascertained via the same case definition as a post-DWH incident case, were excluded from all analyses of that particular neurological outcome.

### Statistical analyses

For our main analyses, we included ICD-9 codes in any diagnostic position. We conducted multivariable Cox Proportional Hazards regression analyses to model associations between the oil spill exposures described above and risk of neurological diseases/symptoms by calculating adjusted hazard ratios (aHRs) and 95% confidence intervals (95% CI). The within-responder comparisons (ever vs. never crude oil exposure metrics and combined crude oil/dispersants comparisons) were adjusted for age at baseline (years), sex (male, female), race (white, Black, other/unknown), and smoking status (never, former, current, unknown) based on prior literature [40, 44]. The responder vs. non-responder models were adjusted only for age, sex, and race because smoking information was not available for non-responders or for responders who did not complete a post-deployment survey (*n*=2472). We calculated p-values for linear trend (p-trend) for the combined oil/dispersants exposure treating it as a continuous variable in the Cox regression models.

For the responder vs. non-responder comparison, the start of follow-up time for all cohort members was the later of April 20, 2010 or the USCG entry date. Responders contributed events and person-time as non-responders until the first day of their spill deployment. Because responders may have sought care outside of the MHS during the DWH spill deployment, their health encounters may have not been recorded in a systematic way. To account for this potential issue, we excluded responder events and person-time during deployment from the study observational period. From the day after their deployment ended, responders contributed events and person-time to the responder group. For the within-responder comparisons, the start of follow-up time for all responders was the day after the last day of their spill deployment. The end of follow-up time for all study comparisons was the earliest of 1) the date of becoming a case of a particular neurological condition, 2) the end of follow-up period, i.e., September 30, 2015, or 3) the USCG exit date.

We tested the assumption of proportionality of hazards across the entire follow-up period (April 20, 2010/end of deployment through September 30, 2015) by evaluating Pearson correlations between Schoenfeld residuals and follow-up time. A *p-*value < 0.05 for the corresponding Pearson correlation coefficient suggested non-proportionality of hazards. In the cases where the proportionality assumption was violated, we calculated aHRs and 95% CIs for two approximately equal-length time periods: April 20, 2010/end of deployment –December 31, 2012 (i.e., the earlier period) and January 1, 2013 –September 30, 2015 (i.e., the later period).

### Sensitivity analyses

To evaluate the robustness of the main associations, we performed four sensitivity analyses. First, we refined incident case definitions by restricting the relevant ICD-9 codes to either the first or the second diagnostic position instead of to *any* diagnostic position. For the second sensitivity analysis, we excluded cohort members who were potentially exposed to occupational hazards and, therefore, under more intensive periodic medical surveillance through enrollment in the Coast Guard’s Occupational Medical Surveillance and Evaluation Program (OMSEP) from before the DWH oil spill through the end of the study follow-up period. USCG members with occupations with a high likelihood for occupational exposure to known or suspected toxins (e.g., benzene) require enrollment in OMSEP and are followed more closely through baseline and periodic physical examinations in accordance with the Occupational Safety and Health Administration (OSHA) requirements [47]. We excluded OMSEP enrollees who were in the program due to occupational exposures to benzene, chromium compounds, lead, pesticides, and/or solvents because these cohort members could be a higher risk group for developing neurological conditions due to their usual occupational exposures and could, therefore, bias our risk estimates for neurological conditions. Because tobacco smoke contains some of the same constituents as crude oil (i.e., benzene, PAHs, heavy metals) [48], for our third sensitivity analysis, we restricted the within-responder comparisons for the ever/never crude oil exposure via inhalation to those responders who reported never smoking. This restriction allowed us to rule out any potential residual confounding by smoking. Lastly, to assess exposure-response relationships between increasing levels of self-reported crude oil inhalation exposure and neurological outcomes, we performed a sensitivity analysis restricted to spill responders with Survey 2 data (*n*=3102).

All analyses were performed in SAS Version 9.4 (SAS Institute, Cary, NC, USA).

## Results

### Baseline population characteristics

Table 1 depicts baseline characteristics of all active duty DWH-CG Cohort members stratified into four groups: 1) all non-responders (*n*= 392630), 2) all responders (*n*=5964), 3) responders with survey data (*n*=3492), and 4) responders without survey data (*n*=2472). The mean baseline age was approximately 30 years regardless of the response status or the survey completion status. Cohort members were predominantly male and white. The proportion of males and white individuals among responders (87.8% and 77.6%, respectively) was slightly higher than among non-responders (85.4% and 76.9%, respectively). Responders without survey data had a slightly higher proportion of males and white members (89.4% and 78.0%, respectively) than responders who completed a post-deployment survey (86.7% and 77.4%, respectively). The proportion of junior enlisted cohort members was the highest among non-responders (56.5%). Responders who did not complete a survey had a slightly higher proportion of junior enlisted members (53.6%) than responders with survey data (48.6%). The majority of cohort members were high school educated. A higher proportion of non-responders (69.8%) than responders (65.3%) and responders without a survey (66.8%) than responders with survey data (64.2%) had been educated through high school. Smoking information was available only for responders who completed a post-deployment survey. The majority (54.1%) reported never smoking, 14.9% reported being former smokers, 22.5% were current smokers, while smoking status of the remaining 8.5% was unknown. The median follow-up time was 5.5 years for non-responders and 5.1 years for responders.

**Table 1:** Characteristics of active duty members of the Deepwater Horizon Oil Spill Coast Guard (DWH-CG) Cohort

Characteristic	Non-responders (*N*=39260)	Responders (*N* =5964)	Responders with survey data (*N* =3492)	Responders with no survey data (*N* = 2472)
**Age (years)**				
Mean (SD)	30.3 (8.2)	30.7 (7.6)	30.9 (7.6)	30.5 (7.6)
**Sex, n (%)**				
Male	33517 (85.4%)	5238 (87.8%)	3028 (86.7%)	2210 (89.4%)
Female	5743 (14.6%)	726 (12.2%)	464 (13.3%)	262 (10.6%)
**Race, n (%)**				
White	30209 (76.9%)	4630 (77.6%)	2703 (77.4%)	1927 (78.0%)
Black	2183 (5.6%)	304 (5.1%)	167 (4.8%)	137 (5.5%)
Asian/AI/AN/NH/PI	1541 (3.9%)	240 (4.0%)	153 (4.4%)	87 (3.5%)
Other	2111 (5.4%)	300 (5.0%)	178 (5.1%)	122 (4.9%)
Unknown	3216 (8.2%)	490 (8.3%)	291 (8.3%)	199 (8.1%)
**Military rank, n (%)**				
Junior enlisted (E1-E5)	22191 (56.5%)	3020 (50.6%)	1696 (48.6%)	1324 (53.6%)
Senior enlisted (E6-E10)	10106 (25.7%)	1454 (24.4%)	868 (24.9%)	586 (23.7%)
Officer (O1-O10, W2-W4)	6963 (17.8%)	1490 (25.0%)	928 (26.5%)	562 (22.7%)
**Highest education, n (%)**				
High school or less	27401 (69.8%)	3893 (65.3%)	2242 (64.2%)	1651 (66.8%)
Some college or higher^a^	10862 (27.7%)	1990 (33.4%)	1203 (34.5%)	787 (31.8%)
Other or not indicated	997 (2.5%)	81 (1.3%)	47 (1.3%)	34 (1.4%)
**Smoking status, n (%)**				
Never	--	--	1888 (54.1%)	--
Former	--	--	521 (14.9%)	--
Current	--	--	786 (22.5%)	--
Missing	--	--	297 (8.5%)	--
**Follow-up time (years)**				
Median	5.5	5.1	5.1	5.2

^a^Some college or higher includes technical school, bachelors, masters, and doctoral degree

*Abbreviations AI* American Indian, *AN* Alaska Native, *NH* Native Hawaiian, *PI* Pacific Islander

### Responder vs. non-responder comparisons

The adjusted hazard ratios (aHRs) for incident neurological diseases/symptoms following the DWH oil spill, comparing all active duty responders to non-responders after adjustment for age, sex, and race, are presented in Table 2. The proportionality of hazards assumption over the study follow-up period (2010-2015) was violated for one of the outcomes (*nerve root and plexus disorders*), as evidenced by a Schoenfeld residual p-value of <0.05. Therefore, we conducted the analyses for this particular outcome separately in the earlier (2010-2012) and in the later time period (2013-2015) (Table 2 footnote). The risk for *nerve root and plexus disorders* comparing responders to non-responders was higher in the earlier time period (aHR=1.29, 95% CI: 0.73-2.29) than in the later time period (aHR=0.75, 95% CI: 0.39-1.45), however neither association reached statistical significance. In the overall follow-up period where the proportionality of hazards assumption was not violated, we found reduced risks for *headache* (aHR=0.84, 95% CI: 0.74-0.96), a fainting condition *syncope and collapse* (aHR=0.74, 95% CI: 0.56-0.97,) and *disturbance of skin sensation* (aHR=0.81, 95% CI: 0.68-0.96) among the responders. The associations between the DWH oil spill response status and all of the other neurological conditions we evaluated were not statistically significant – the general pattern suggested null or reduced risks for responders.

**Table 2:** Risk of neurological conditions comparing active duty DWH-CG Cohort responders to non-responders, 2010-2015

	Responder (***N***=5964)		Non-responder (***N***=39260)		
Condition (ICD-9 code)	N	Person Years	N	Person Years	HR^**a**^ (95% CI)
Migraine (346)	156	25629	1181	172292	0.97 (0.82-1.14)
Migraine with aura (346.0)	30	26462	181	177225	1.15 (0.78-1.70)
Migraine without aura (346.1)	22	26465	234	176852	0.66 (0.43-1.03)
Migraine, unspecified (346.9)	130	25812	933	173573	1.03 (0.86-1.24)
Migraine excl. menstrual migraine and persistent migraine aura with cerebral infarction (346.1-346.3, 346.5, 346.7-346.9)	145	25704	1097	172660	0.97 (0.81-1.15)
Headache (784.0)	245	25244	2047	167802	**0.84 (0.74-0.96)**
Other headache syndromes (339)	81	26313	557	176064	0.99 (0.79-1.26)
Headaches/migraines combined (339, 346, 784.0)	371	24383	2926	163050	0.91 (0.82-1.01)
Headaches/migraines combined excl. menstrual migraine and persistent migraine aura with cerebral infarction (339, 346.1-346.3, 346.5, 346.7-346.9, 784.0)	366	24438	2872	163325	0.92 (0.82-1.02)
Memory loss (780.93)	21	26538	154	177431	0.88 (0.55-1.38)
Signs and symptoms involving cognition (799.5)	13	26564	65	177742	1.24 (0.68-2.25)
Attention or concentration deficit (799.51)	10	26570	52	177757	1.19 (0.61-2.34)
Essential and other specified forms of tremor (333.1)	10	26565	56	177618	1.19 (0.60-2.33)
Restless legs syndrome (333.94)	27	26499	200	177262	0.89 (0.59-1.33)
Facial nerve disorders (351)	15	26511	83	177474	1.23 (0.71-2.14)
Nerve root and plexus disorders (353)	24	26488	159	177262	1.00 (0.65-1.53)^b^
Mononeuritis of upper limb and mononeuritis multiplex (354)	123	26135	808	174996	1.02 (0.84-1.23)
Carpal tunnel syndrome (354.0)	81	26309	519	176051	1.06 (0.83-1.34)
Mononeuritis of lower limb (355)	74	26397	470	176353	1.03 (0.80-1.31)
Peripheral neuropathy (356.4, 356.8, 356.9, 357.89)	17	26520	143	177384	0.78 (0.47-1.30)
Visual disturbances (368)	87	26200	647	175307	0.90 (0.72-1.13)
Subjective visual disturbances (368.1)	21	26512	114	177400	1.27 (0.79-2.02)
Hearing loss (389)	292	25461	1734	171674	1.10 (0.97-1.24)
Tinnitus (388.3)	131	26198	948	175226	0.89 (0.74-1.07)
Syncope and collapse (780.2)	57	26298	531	175386	**0.74 (0.56-0.97)**
Dizziness and giddiness (780.4)	146	25950	996	173680	1.00 (0.84-1.19)
Abnormality of gait (781.2)	66	26326	395	176294	1.09 (0.84-1.42)
Disturbance of skin sensation (782.0)	146	26025	1197	173279	**0.81 (0.68-0.96)**

^a^Models adjusted for age, sex, and race

^b^Because of the proportionality of hazards assumption violation for *nerve root and plexus disorders* during 2010-2015 (Schoenfeld *p*<0.05), results from sub-period analyses were: 2010-2012: N_responder_=14, N_non-responder_=79, HR=1.29, 95% CI: 0.73-2.29 and 2013-2015: N_responder_ =10, N_non-responder_ =80, HR=0.75, 95% CI: 0.39-1.45.

**Bold** indicative of statistical significance

In the sensitivity analysis where we refined the neurological case definitions by restricting cases to those with ICD-9 codes in either the first or the second diagnostic position, instead of in *any* diagnostic position, we observed similar patterns and magnitudes of associations for most of the outcomes (Supplemental Table 2). While the estimates for *syncope and collapse* and *disturbance of skin sensation* were similar to the original estimates in Table 2, they were no longer significantly reduced in this sensitivity analysis.

After exclusion of 1169 (3.0%) non-responders and 242 (4.1%) responders who were enrolled in the Coast Guard’s surveillance program OMSEP during the follow-up period (Supplemental Table 3), the patterns of risk did not change.

### Within-responder comparisons: crude oil inhalation

For all of the within-responder comparisons, we present a smaller number of neurological outcomes for which there were at least nine cases per exposure group in the overall follow-up period (2010-2015).

In Table 3 we show age-, sex-, race-, and smoking-adjusted HRs and 95% CIs, comparing active duty responders who reported *ever* exposure to crude oil via inhalation to those responders who reported *never* crude oil inhalation exposure. In the overall follow-up period, where the proportionality of hazards assumption was not violated, we observed elevated risks for several individual and grouped outcomes related to headaches and migraines, including *headache* (aHR=1.47, 95% CI: 1.07-2.04), *other headache syndromes* (aHR=1.83, 95% CI: 1.03-3.25), a grouped set of conditions *headaches/migraines combined* (aHR=1.41, 95% CI: 1.08-1.84), and *headaches/migraines combined excluding menstrual migraine and persistent migraine aura with cerebral infarction* (aHR=1.39, 95% CI: 1.06-1.81). There was also a suggestion of an elevated risk for *migraine with aura* (aHR=2.22, 95% CI: 0.98-5.01). Crude oil inhalation exposure was also associated with elevated risks for an inflammatory nerve condition *mononeuritis of upper limb and mononeuritis multiplex* (aHR=1.71, 95% CI: 1.04-2.83), and *tinnitus* (aHR=1.91, 95% CI: 1.23-2.96), a condition defined by a ringing or buzzing noise in one or both ears. For one of the neurological conditions, *syncope and collapse*, the proportionality of hazards assumption was violated, as evidenced by the Schoenfeld p-value of <0.05, thus, we performed period-specific analyses. We had insufficient number of *syncope and collapse* incident cases in the earlier time period to meaningfully evaluate the association, nonetheless, the risk for *syncope and collapse* was suggestively elevated (aHR=2.35, 95% CI: 0.98-5.63) in the later time period (Table 3 footnote).

**Table 3:** Risk of neurological conditions among active duty DWH-CG Cohort responders reporting ever vs. never exposure to crude oil inhalation, 2010-2015

	Oil inhalation ever (*N=*1068)		Oil inhalation never (*N*=2424)		
Condition (ICD-9 code)	N	Person Years	N	Person Years	HR^a^ (95% CI)
Migraine (346)	37	4711	65	10426	1.41 (0.93-2.13)
Migraine with aura (346.0)	11	4874	14	10787	2.22 (0.98-5.01)
Migraine, unspecified (346.9)	26	4762	60	10490	1.05 (0.66-1.68)
Migraine excl. menstrual migraine and persistent migraine aura with cerebral infarction (346.1-346.3, 346.5, 346.7-346.9)	34	4725	60	10454	1.38 (0.90-2.11)
Headache (784.0)	62	4616	100	10259	**1.47 (1.07-2.04)**
Other headache syndromes (339)	21	4831	30	10731	**1.83 (1.03-3.25)**
Headaches/migraines combined (339, 346, 784.0)	90	4444	157	9906	**1.41 (1.08-1.84)**
Headaches/migraines combined excl. menstrual migraine and persistent migraine aura with cerebral infarction (339, 346.1-346.3, 346.5, 346.7-346.9, 784.0)	88	4456	156	9922	**1.39 (1.06-1.81)**
Mononeuritis of upper limb and mononeuritis multiplex (354)	27	4805	42	10675	**1.71 (1.04-2.83)**
Carpal tunnel syndrome (354.0)	16	4851	28	10727	1.51 (0.80-2.84)
Mononeuritis of lower limb (355)	15	4871	30	10761	1.22 (0.65-2.30)
Visual disturbances (368)	18	4814	38	10674	1.11 (0.62-1.96)
Hearing loss (389)	52	4679	123	10383	1.05 (0.75-1.46)
Tinnitus (388.3)	37	4825	50	10680	**1.91 (1.23-2.96)**
Syncope and collapse (780.2)	13	4858	29	10699	1.12 (0.57-2.19)^b^
Dizziness and giddiness (780.4)	20	4829	68	10536	0.73 (0.44-1.22)
Abnormality of gait (781.2)	13	4860	24	10736	1.17 (0.59-2.32)
Disturbance of skin sensation (782.0)	31	4804	61	10598	1.13 (0.73-1.76)

^a^Models adjusted for age, sex, race, and smoking; ^b^ Because of the proportionality of hazards assumption violation for *syncope and collapse* during 2010-2015 (Schoenfeld p<0.05), results from sub-period analyses were: 2010-2012: N_oil inhal ever_=3, N_oil inhal never_=17, HR=0.41, 95% CI: 0.12-1.42 and 2013-2015: N_oil inhal ever_ =10, N_oil inhal never_ =12, HR 2.35, 95% CI: 0.98-5.63.

**Bold** indicative of statistical significance

In the sensitivity analysis restricting neurological cases to ICD-9 codes in either the first or the second diagnostic position (Supplemental Table 4), patterns of risk remained generally similar to the main analysis presented in Table 3, with an exception for the neurological outcome *tinnitus* for which the proportionality of hazards assumption was violated in the overall time period. There was an insufficient number of *tinnitus* cases in the earlier time period to evaluate the aHR (Supplemental Table 4 footnote), while the risk was elevated in the later time period (aHR=2.15, 95% CI: 1.17-3.96) among responders reporting oil inhalation exposure.

The sensitivity analysis excluding 152 (4.4%) responders enrolled in OMSEP during the study follow-up period (Supplemental Table 5) was largely similar to the main analysis, with the exception of *other headache syndromes* for which the adjusted HR (95% CI) estimate attenuated to 1.68 (0.93-3.05).

In a separate sensitivity analysis restricted to 54.1% of responders who reported never smoking (Supplemental Table 6), patterns of risks in the overall follow-up period were generally similar to the main analysis, however, the associations with most headaches and migraines outcomes attenuated (i.e., *headache* aHR=1.36, 95% CI: 0.85-2.17, *other headache syndromes* aHR=1.74, 95% CI: 0.74-4.12, *headaches/migraines combined* aHR=1.35, 95% CI: 0.94-1.94, and *headaches/migraines combined excluding menstrual migraine and persistent migraine aura with cerebral infarction* aHR=1.35, 95% CI: 0.93-1.94), except for *migraine with aura* for which the association strengthened and became statistically significant (aHR=2.71, 95% CI: 1.06-6.91). The risk for *mononeuritis of upper limb and mononeuritis multiplex* also strengthened (aHR=2.08, 95% CI: 1.04-4.18). Additionally, the proportionality of hazards assumption was violated for *tinnitus*. While there was not a sufficient number of *tinnitus* cases in the earlier time period to evaluate the association, the risk for *tinnitus* was elevated in the later time period (aHR=3.37, 95% CI: 1.58-7.18).

In the last sensitivity analysis, restricted to the 3102 responders with Survey 2 data, we examined associations between the crude oil inhalation exposure assessed on a 5-point Likert scale and, due to statistical power, a limited number of neurological conditions (Supplemental Table 7). Increasing levels of crude oil inhalation exposure were associated with increasing risks for *headache* (aHR _rarely vs. never_=1.11, aHR _sometimes vs. never_=1.28, aHR _most of the time vs. never_=1.62, aHR _all of the time vs. never_=1.97, p-trend=0.02), *headaches/migraines combined* (aHR _rarely vs. never_=1.13, aHR _sometimes vs. never_=1.35, aHR _most of the time vs. never_=1.57, aHR _all of the time vs. never_=2.05, p-trend=0.003), *mononeuritis of upper limb and mononeuritis multiplex* (aHR _rarely vs. never_=1.41, aHR _sometimes vs. never_=1.44, aHR _most of the time vs. never_=2.18, aHR _all of the time vs. never_=3.84, p-trend=0.008), *and tinnitus* (aHR _rarely vs. never_=1.48, aHR _sometimes vs. never_=2.52, aHR _most of the time vs. never_=1.93, aHR _all of the time vs. never_=2.51, p-trend=0.004).

### Within-responder comparisons: other crude oil exposures

The results for the ever vs. never crude oil exposure via any route (i.e., inhalation, direct skin contact, ingestion, or submersion) metric are presented in Supplemental Table 8. The patterns and magnitudes of these associations were generally similar to the crude oil inhalation results presented in Table 3. The results for the crude oil exposure via direct skin contact and via submersion were also similar (data not shown).

### Within-responder comparisons: combined crude oil and dispersants exposure

The age-, sex-, race-, and smoking-adjusted associations of self-reported exposures to “Oil only” and to both crude oil and dispersants (“Both”) compared to neither exposure are presented in Table 4. The proportionality of hazards assumption was not violated for any of the outcomes in the overall follow-up period. The associations for all of the neurological conditions, except for the *disturbance of skin sensation*, followed the same trend of greater magnitude of risk among responders reporting exposure to both crude oil and dispersants (vs. neither) than among responders reporting “Oil only” (vs. neither) exposure. The risk for *headache* in the overall follow-up period was significantly elevated for the exposure to both crude oil and dispersants (aHR=1.63, 95% CI: 1.03-2.57), but non-significantly elevated for the “Oil only” exposure (aHR=1.12, 95% CI: 0.77-1.62, p-trend=0.06). We observed a similar trend of elevated risks for *headaches/migraines combined* (aHR _both vs. neither_ = 1.69, 95% CI: 1.17-2.45; aHR _oil only vs. neither_ =1.20, 95% CI: 0.89-1.62, p-trend=0.008), *headaches/migraines combined excluding menstrual migraine and persistent migraine aura with cerebral infarction* (aHR _both vs. neither_ = 1.70, 95% CI: 1.17-2.47; aHR _oil only vs. neither_ =1.25, 95% CI: 0.93-1.68, p-trend=0.006), *mononeuritis of upper limb and mononeuritis multiplex* (aHR _both vs. neither_ = 2.71, 95% CI: 1.34-5.49; aHR _oil only vs. neither_ =1.61, 95% CI: 0.89-2.91, p-trend=0.006), and *tinnitus* (aHR _both vs. neither_ = 2.22, 95% CI: 1.14-4.31; aHR _oil only vs. neither_ =1.65, 95% CI: 0.96-2.82, p-trend=0.01).

**Table 4:** Risk of neurological conditions among active duty DWH-CG Cohort responders reporting exposure to oil only (*N*=1351), both crude oil and dispersant (*N*=448) vs. neither exposure (*N*=1283), 2010-2015

Condition (ICD-9 code)	N	Person Years	HR^a^ (95% CI)	p-trend
**Migraine (346)**				
Neither	36	5595	1.00	
Oil only	39	5886	1.12 (0.71-1.78)	
Both	19	1939	1.74 (0.99-3.05)	0.08
**Migraine, unspecified (346.9)**				
Neither	32	5641	1.00	
Oil only	33	5930	1.07 (0.65-1.75)	
Both	15	1960	1.55 (0.83-2.89)	0.22
**Migraine excl. menstrual migraine and persistent migraine aura with cerebral infarction** **(346.1-346.3, 346.5, 346.7-346.9)**				
Neither	32	5614	1.00	
Oil only	40	5898	1.29 (0.80-2.06)	
Both	16	1948	1.63 (0.89-2.99)	0.10
**Headache (784.0)**				
Neither	54	5492	1.00	
Oil only	62	5796	1.12 (0.77-1.62)	
Both	29	1920	**1.63 (1.03-2.57)**	0.06
**Headaches/migraines combined (339, 346, 784.0)**				
Neither	83	5327	1.00	
Oil only	97	5580	1.20 (0.89-1.62)	
Both	44	1850	**1.69 (1.17-2.45)**	**0.008**
**Headaches/migraines combined excl. menstrual migraine and persistent migraine aura with cerebral infarction** **(339, 346.1-346.3, 346.5, 346.7-346.9, 784.0)**				
Neither	81	5336	1.00	
Oil only	98	5593	1.25 (0.93-1.68)	
Both	43	1852	**1.70 (1.17-2.47)**	**0.006**
**Mononeuritis of upper limb and mononeuritis multiplex (354)**				
Neither	19	5723	1.00	
Oil only	28	6010	1.61 (0.89-2.91)	
Both	14	2007	**2.71 (1.34-5.49)**	**0.006**
**Hearing loss (389)**				
Neither	64	5560	1.00	
Oil only	64	5814	1.02 (0.72-1.45)	
Both	25	1970	1.23 (0.77-1.97)	0.46
**Tinnitus (388.3)**				
Neither	22	5709	1.00	
Oil only	36	6060	1.65 (0.96-2.82)	
Both	15	2002	**2.22 (1.14-4.31)**	**0.01**
**Disturbance of skin sensation (782.0)**				
Neither	36	5645	1.00	
Oil only	39	5997	1.01 (0.64-1.59)	
Both	11	2004	0.89 (0.45-1.75)	0.79

^a^Models adjusted for age, sex, race, and smoking; **Bold** indicative of statistical significance

## Discussion

In this prospective study of young and generally healthy active duty U.S. Coast Guard service members with comprehensive military healthcare coverage, we found that self-reported exposures to crude oil inhalation and to combined crude oil and dispersants during the spill cleanup were associated with increased risks for diagnoses of several neurological conditions during the five and a half years following the *Deepwater Horizon* oil spill. Compared to non-exposed responders, those who reported a crude oil inhalation exposure were at approximately 40 to 80% increased risk for developing different headache- and migraine-related conditions. Responders with reported crude oil inhalation exposure were also at 70% elevated risk for being diagnosed with an inflammatory nerve condition, *mononeuritis of upper limb and mononeuritis multiplex*, and at 90% increased risk for a diagnosis of *tinnitus*, a condition characterized by ringing in one or both ears. Risk estimates for those neurological conditions were higher in magnitude among responders reporting exposure to both crude oil and dispersants compared to those reporting neither exposure than among responders reporting exposure to crude oil only (vs. neither exposure). Patterns of risks remained robust across a range of sensitivity analyses, and evidence of an exposure-response trend was found for several of the conditions with sufficient statistical power for categorizing by five levels of crude oil inhalation exposure. Compared to not responding to the spill, a crude measure of exposure of responding to the spill was associated with decreased risks of being diagnosed with *headache*, *syncope and collapse*, and *disturbance of skin sensation*.

Our finding of elevated risks for headache- and migraine-related conditions is in agreement with prior findings from cross-sectional studies of acute symptoms during or shortly after oil spill cleanup participation [28, 30, 32–34, 36–40]. For instance, in a comprehensive cross-sectional study conducted among the USCG responders to the DWH oil spill, increasing frequency of self-reported crude oil exposure via inhalation (rarely, sometimes, most/all times vs. never) was associated with increased reports of headaches (adjusted prevalence ratio, aPR_most/all vs. never_=1.80, 95% CI: 1.61-2.01, p-trend <0.01) [42]. Additionally, in agreement with our prospective findings, prevalence of headaches was higher in magnitude among responders with reported exposure to both crude oil and dispersants (vs. neither) than among those who were exposed to crude oil, but not to dispersants (vs. neither exposure) [42]. To our knowledge, there has only been one other study to date, also conducted by our study team, that evaluated risk of headaches/migraines up to two years post-DWH spill among active duty USCG responders [40]. In that study, compared to responders who reported no exposure to crude oil via *any* route, the crude oil-exposed responders had a non-significantly elevated risk for *headaches and migraines* diagnoses (ICD-9 codes 339, 346; aRR=1.35, 95% CI: 0.95-1.92) [40]. In the present study, we expanded upon our previous investigation by including a longer follow-up of 5.5 years, more headache- and migraine-related outcomes, and more specific exposure metrics including crude oil inhalation and a combined crude oil and dispersants exposure, and observed significantly elevated risks for several headache and migraine diagnoses. Of note, although not reaching statistical significance, spill responders reporting crude oil inhalation exposure had an elevated risk for *migraine with aura* (aHR=2.22, 95% CI: 0.98-5.01) in the overall analysis (Table 3) and the association strengthened and reached statistical significance (aHR=2.71, 95% CI: 1.06-6.91) in the sensitivity analysis restricted to never-smokers (Supplemental Table 6).  Given that headaches have been associated with exposure to crude oil constituents (e.g., VOCs [7–11, 13] and hydrogen sulfide [14]) and to 2-butoxyethanol [17, 18], a constituent of oil dispersant COREXIT 9527A, our findings are plausible.

To our knowledge, the only other study that evaluated longer-term neurological outcomes in relation to oil spill cleanup exposures was recently conducted among the GuLF Study participants four to six years after their DWH oil spill response [44]. In that study, Quist et al. compared 16 neurobehavioral function scores assessing attention, memory, executive function, response speed, coordination, and effort-related motivation across different cleanup jobs (i.e., cleanup on land, decontamination, cleanup on water, operations, and response work vs. administrative support) and across estimated total hydrocarbon (THC) exposure levels (0.30-0.99, 1.0-2.99, and ≥3 vs. <0.30 ppm) [44]. Both cleanup jobs with higher oil spill exposure opportunities (compared to administrative support work), and higher estimated THC levels (compared to lower levels) were modestly associated with impairments in attention and memory, executive function, working memory, effort-related motivation, and response speed/coordination [44]. While our study did not assess neurobehavioral function, we did observe associations between self-reported crude oil and dispersant exposures and increased risk of *mononeuritis of upper limb and mononeuritis multiplex*, a type of peripheral neuropathy that can also potentially impair performance on the symbol digit test and response speed/coordination due to a loss of sensation or weakness in one or more upper limb nerves. In fact, in our previous cross-sectional study of acute symptoms experienced during the oil spill response, increasing frequency of self-reported crude oil inhalation exposure was associated with increased reports of a numbness/tingling sensation (aPR_most/all vs. never_ =3.32, 2.19-5.05, p-tend<0.01) [42]. In a different study conducted among a convenience sample of 690 Gulf residents enrolled in the GuLF Study, blood BTEX concentrations were measured two to three years after the DWH cleanup participation and associations between BTEX levels and a cluster of PNS symptoms, including tingling and numbness in the extremities, blurred vision, and stumbling while walking, were examined [43]. In an analysis restricted to non-smokers and adjusted for chemical co-exposures, the highest quartile of toluene exposure was significantly associated with multiple PNS symptoms (aPR=3.11, 95% CI: 1.13-8.52, p-trend<0.05) [43]. While the participants’ blood BTEX levels were unlikely to be due to their DWH spill cleanup response work (due to the short biological half-lives of BTEX), but rather to the background petrochemical industry exposures among the Gulf residents [43], the GuLF Study participants also had an opportunity to be exposed to the BTEX chemicals via crude oil exposures during the spill cleanup response work.

Our finding of approximately two-fold increased risk of *tinnitus* among responders reporting crude oil inhalation and among responders reporting both crude oil and dispersant exposure is novel. While we are not aware of any other studies in the oil spill literature investigating *tinnitus*, we believe that our finding is plausible given the potential mechanism of ototoxicity related to exposure to VOC chemicals toluene [8] and ethylbenzene [11]. Ringing in the ears, or tinnitus, may be one of the first signs of ototoxicity, which may eventually progress to hearing loss. While we also investigated risk of *hearing loss*, the association with crude oil exposure was not significant. Occupational noise exposure is one of the main risk factors for tinnitus. Because exposure to noise is one of the reasons for enrolling in the Coast Guard’s occupational surveillance program OMSEP, we investigated whether the association between crude oil inhalation exposure and risk of *tinnitus* was modified by OMSEP enrollment status due to occupational noise exposure. Among 791 (22.7%) responders who were enrolled in the OMSEP hearing protection program due to occupational noise exposure, the self-reported crude oil inhalation exposure was non-significantly associated with elevated risk of *tinnitus* (aHR=1.81, 95% CI: 0.78-4.20). Among the remaining 2701 responders who were not in the OMSEP hearing protection program, the association between crude oil inhalation and *tinnitus* was similar and statistically significant (aHR=1.77, 95% CI: 1.06-2.94, p-interaction=0.94). Therefore, we did not find that the association between crude oil inhalation exposure and elevated risk of *tinnitus* was modified by the responders’ occupational noise exposure during their career in the Coast Guard.

In the age-, sex, and race-adjusted analyses based on the more crude exposure metric of spill response status (responder vs. non-responder), we found that oil spill response was associated with reduced risks for *headache*, *syncope and collapse*, and *disturbance of skin sensation*. However, in the within-responder analyses, which were further adjusted for smoking and based on a more specific exposure metric of crude oil inhalation, the risk for *headache* was significantly elevated among the exposed. There was also a suggestion of elevated risk for *syncope and collapse* in the later time period (Table 3 footnote), and a non-significantly elevated risk for *disturbance of skin sensation* in the overall time period among the exposed. This paradox could be due to a healthy worker effect (i.e., a healthy deployer bias) in the responder vs. non-responder analyses. At the time of the DWH oil spill, the USCG did not have a centralized database of personnel who were not fit for deployment due to different reasons including injury, pregnancy, or awaiting medical clearance for various conditions. We were therefore not able to exclude any non-responders who may have been medically unfit for deployment. This could have affected up to 10% of our non-responder comparison population based on recent U.S. military estimates [49], a figure likely similar to the USCG estimates of personnel not fit for deployment (personal communication with Dana L. Thomas on March 14, 2021). It is also possible that the reduced risks we observed could have been a result of a bias arising from inclusion of both exposed and non-exposed individuals among spill responders.

Our study has several strengths. The large sample size of our cohort allowed us to assess the robustness of our findings through various sensitivity analyses. We used several metrics of exposure, including crude oil inhalation and a combined crude oil and dispersants exposure, to further elucidate human health effects associated with realistic exposure scenarios of oil spill cleanup participation. To our knowledge, our study was the first to ascertain the incidence of longer-term neurological outcomes from an objective and comprehensive database of health encounters, thus decreasing the potential for recall errors in disease ascertainment. Being part of a universal healthcare system designed for equal access and coverage likely also reduced the potential for selection bias and differential loss to follow-up. Because we had access to health information before the DWH oil spill, we were able to exclude USCG members with pre-existing conditions and evaluate incident neurological disease and symptoms. Since our cohort consisted of young and relatively healthy active duty service members, the likelihood of existing co-morbidities was low.

One of the main limitations of our study is related to the lack of individual-level occupational monitoring data. Our main exposure metrics were based on self-reported survey data which are prone to recall errors. However, the DWH responders completed exit surveys relatively shortly after end of deployment (i.e., a median of one day for Survey 1 and 153 days for Survey 2) [40] and any potential recall error is likely to be non-differential. The average age of our generally healthy population at the beginning of the five and a half year follow-up period was approximately 30 years. This follow-up duration was not long enough for young people to develop neurological disorders with a long latency period, such as neurodegenerative conditions including Parkinson’s disease. Therefore, a large part of our investigation was limited to evaluating relatively non-specific outcomes including headaches, *syncope and collapse*, *dizziness and giddiness,* and *abnormality of gait*. Some of the conditions we evaluated were also not necessarily neurological in nature (e.g., visual disturbance, disturbance of skin sensation), but could be symptoms of neurological disturbances. Nevertheless, some of these outcomes and symptoms may be risk factors for, or precursors to, more severe neurological diseases as our cohort ages. Studies with a long-term follow-up are needed to evaluate associations between oil spill exposures and neurological diseases with a long latency period. Because we carried out multiple comparisons across a few exposures and several neurological outcomes, some of our results may be statistically significant due to chance, although most of our significant findings were confirmed in sensitivity analyses. Additionally, given the paucity of research on longer-term neurological impacts of oil spill response exposures, our primary goal was to evaluate patterns of association rather than to test any specific hypothesis. While our neurological outcomes were defined using objectively ascertained military health encounter data, ICD coding may be susceptible to classification inaccuracies such as coder errors and differences in electronic medical records across facilities [50]. Nonetheless, ICD coding is generally a reliable indicator of disease/symptoms diagnoses when interpreted with caution, and as a result has been used widely in epidemiological research [50] and military surveillance [51]. In order to increase diagnostic accuracy of ICD-9 coding classifications in our own analyses, our incident case definitions required one inpatient or two outpatient visits. To further refine case definitions, we additionally conducted sensitivity analyses limiting the ICD-9 codes to the first or second diagnostic position. Finally, because our study population was largely white, male, young, and generally healthy, our findings may not be generalizable to all oil spill responders.

## Conclusions

In this large study of active duty USCG personnel with universal military healthcare coverage, we found that *Deepwater Horizon* cleanup exposures were moderately associated with increased risks for longer-term neurological conditions. As aggressive expansion of deepwater exploration and drilling continues [2, 52] and offshore drilling regulations become more lax [2], oil spill disasters will continue to occur and affect the ecosystem, wildlife, and human health. Given the frequency and volume of oil spill disasters, their frequent occurrence in environmentally sensitive areas already under threat by climate change, as well as our limited knowledge of long-term health consequences of oil spills, it is of critical public health importance to continue studying potential long-term adverse health outcomes of oil spill response workers and individuals residing in communities affected by oil spills. Our study findings may inform disaster preparedness officials of potential preventative and mitigation strategies needed to support responders to future oil spill disasters.

## Supplementary Information

**Additional file 1.**

## Data Availability

Since these data are from military data resources, the process for obtaining assurances for public use requires authorizations not only by the Department of Defense, but also by the U.S. Coast Guard. The corresponding author can provide additional information on the process upon request.

## Abbreviations

aHR: Adjusted hazard ratio
BTEX: Benzene, toluene, ethylbenzene, and xylenes
CI: Confidence interval
CNS: Central nervous system
DoD: Department of Defense
DWH: Deepwater Horizon
DWH-CG Cohort Study: The Deepwater Horizon Coast Guard Cohort Study
GuLF Study: The Gulf Long Term Follow-up Study
HR: Hazard ratio
ICD: The International Classification of Diseases
IRB: The Institutional Review Board
MDR: The Military Health System Data Repository
MHS: The Military Health System
OMSEP: Occupational Medical Surveillance and Evaluation Program
OSHA: The Occupational Safety and Health Administration
PAH: Polycyclic aromatic hydrocarbon
PNS: Peripheral nervous system
PR: Prevalence ratio
THC: Total hydrocarbons
USCG: U.S. Coast Guard
USU: The Uniformed Services University
VOC: Volatile organic compound

## References

[1] Federal On Scene Coordinator (2011). On Scene Coordinator Report: Deewpater Horizon Oil Spill.

[2] Graham B, Reilly W, Beinecke F (2011). Deep water: The gulf oil disaster and the future of offshore drilling.

[3] Berenshtein I, Paris CB, Perlin N, Alloy MM, Joye SB, Murawski S (2020). Invisible oil beyond the Deepwater Horizon satellite footprint. Sci Adv..

[4] McNutt MK, Camilli R, Crone TJ (2012). Review of flow rate estimates of the Deepwater Horizon oil spill. Proc Natl Acad Sci U S A..

[5] Crone TJ, Tolstoy M (2010). Magnitude of the 2010 Gulf of Mexico oil leak. Science..

[6] Institute of Medicine. Assessing the Effects of the Gulf of Mexico Oil Spill on Human Health: A Summary of the June 2010 Workshop. Washington, D.C.; 2010. 1879-0038 (Electronic)

[7] Agency for Toxic Substances and Disease Registry (ATSDR) (2007). Toxicological Profile for Benzene.

[8] Agency for Toxic Substances and Disease Registry (ATSDR) (2015). Toxicological Profile for Toluene.

[9] Win-Shwe TT, Fujimaki H (2010). Neurotoxicity of toluene. Toxicol Lett..

[10] Filley CM, Halliday W, Kleinschmidt-DeMasters BK (2004). The effects of toluene on the central nervous system. J Neuropathol Exp Neurol..

[11] Agency for Toxic Substances and Disease Registry (ATSDR) (2010). Toxicological Profile for Ethylbenzene.

[12] Agency for Toxic Substances and Disease Registry (ATSDR). Toxicological profile for Xylene. Atlanta: U.S. Department of Health and Human Services, Public Health Service; 2007. 38412207

[13] McKee RH, Lammers JH, Muijser H, Owen DE, Kulig BM (2010). Neurobehavioral effects of acute exposure to aromatic hydrocarbons. Int J Toxicol..

[14] Agency for Toxic Substances and Disease Registry (ATSDR) (2006). Toxicological profile for Hydrogen Sulfide.

[15] Agency for Toxic Substances and Disease Registry (ATSDR) (2004). Toxicological profile for Copper.

[16] Agency for Toxic Substances and Disease Registry (ATSDR) (2020). Toxicological profile for Lead.

[17] Agency for Toxic Substances and Disease Registry (ATSDR) (1998). Toxicological profile for 2-butoxyethanol and 2-butoxyethanol acetate.

[18] Agency for Toxic Substances and Disease Registry (ATSDR) (2010). Oil Spill Dispersant (COREXIT ®EC9500A and EC9527A) Information for Health Professionals.

[19] Aguilera F, Mendez J, Pasaro E, Laffon B (2010). Review on the effects of exposure to spilled oils on human health. J Appl Toxicol..

[20] Laffon B, Pasaro E, Valdiglesias V (2016). Effects of exposure to oil spills on human health: Updated review. J Toxicol Environ Health B Crit Rev..

[21] Campbell D, Cox D, Crum J, Foster K, Christie P, Brewster D (1993). Initial effects of the grounding of the tanker Braer on health in Shetland. BMJ..

[22] Campbell D, Cox D, Crum J, Foster K, Riley A (1994). Later effects of grounding of tanker Braer on health in Shetland. BMJ..

[23] Crum JE (1993). Peak expiratory flow rate in schoolchildren living close to Braer oil spill. BMJ..

[24] Lyons RA, Temple JM, Evans D, Fone DL, Palmer SR (1999). Acute health effects of the Sea Empress oil spill. J Epidemiol Community Health..

[25] Janjua NZ, Kasi PM, Nawaz H (2006). Acute health effects of the Tasman Spirit oil spill on residents of Karachi, Pakistan. BMC Public Health.

[26] Kim BM, Park E, LeeAn SY (2009). BTEX exposure and its health effects in pregnant women following the Hebei Spirit oil spill. J Prev Med Public Health..

[27] Alexander M, Engel LS, Olaiya N (2018). The deepwater horizon oil spill coast guard cohort study: A cross-sectional study of acute respiratory health symptoms. Environ Res..

[28] Carrasco J, Lope V, Perez-Gomez B (2006). Association between health information, use of protective devices and occurrence of acute health problems in the Prestige oil spill clean-up in Asturias and Cantabria (Spain): a cross-sectional study. BMC Public Health..

[29] Meo SA, Al-Drees AM, Meo IM, Al-Saadi MM, Azeem MA (2008). Lung function in subjects exposed to crude oil spill into sea water. Mar Pollut Bull..

[30] Na JU, Sim MS, Jo IJ, Song HG (2012). The duration of acute health problems in people involved with the cleanup operation of the Hebei Spirit oil spill. Mar Pollut Bull..

[31] Peres LC, Trapido E, Rung AL (2016). The Deepwater Horizon Oil Spill and Physical Health among Adult Women in Southern Louisiana: The Women and Their Children's Health (WaTCH) Study. Environ Health Perspect..

[32] Suarez B, Lope V, Perez-Gomez B (2005). Acute health problems among subjects involved in the cleanup operation following the Prestige oil spill in Asturias and Cantabria (Spain). Environ Res..

[33] Morita A, Kusaka Y, Deguchi Y (1999). Acute health problems among the people engaged in the cleanup of the Nakhodka oil spill. Environ Res..

[34] Schvoerer C, Gourier-Frery C, Ledrans M (2000). Epidemiologic study on short-term health alterations in people participating in the cleanup of places contaminated by Erika oil (in French).

[35] Meo SA, Al-Drees AM, Rasheed S (2009). Effect of duration of exposure to polluted air environment on lung function in subjects exposed to crude oil spill into sea water. Int J Occup Med Environ Health..

[36] Meo SA, Al-Drees AM, Rasheed S (2009). Health complaints among subjects involved in oil cleanup operations during oil spillage from a Greek tanker "Tasman Spirit". Int J Occup Med Environ Health..

[37] Sim MS, Jo IJ, Song HG (2010). Acute health problems related to the operation mounted to clean the Hebei Spirit oil spill in Taean, Korea. Mar Pollut Bull..

[38] Cheong HK, Ha M, Lee JS (2011). Hebei spirit oil spill exposure and subjective symptoms in residents participating in clean-up activities. Environ Health Toxicol..

[39] Ha M, Kwon H, Cheong HK (2012). Urinary metabolites before and after cleanup and subjective symptoms in volunteer participants in cleanup of the Hebei Spirit oil spill. Sci Total Environ.

[40] Rusiecki J, Alexander M, Schwartz EG (2017). The Deepwater Horizon Oil Spill Coast Guard Cohort study. Occup Environ Med..

[41] McGowan CJ, Kwok RK, Engel LS, Stenzel MR, Stewart PA, Sandler DP (2017). Respiratory, Dermal, and Eye Irritation Symptoms Associated with Corexit EC9527A/EC9500A following the Deepwater Horizon Oil Spill: Findings from the GuLF STUDY. Environ Health Perspect..

[42] Krishnamurthy J, Engel LS, Wang L (2019). Neurological symptoms associated with oil spill response exposures: Results from the Deepwater Horizon Oil Spill Coast Guard Cohort Study. Environ Int..

[43] Werder EJ, Engel LS, Blair A, Kwok RK, McGrath JA, Sandler DP (2019). Blood BTEX levels and neurologic symptoms in Gulf states residents. Environ Res..

[44] Quist AJL, Rohlman DS, Kwok RK (2019). Deepwater Horizon oil spill exposures and neurobehavioral function in GuLF study participants. Environ Res..

[45] Rusiecki JA, Denic-Roberts H, Thomas DL, et al. Incidence of chronic respiratory conditions among oil spill responders: Five years of follow-up in the deepwater horizon oil Spill Coast Guard Cohort study. Environ Res. 2021;111824. 10.1016/j.envres.2021.111824 PMC8616774 34364859

[46] Rhon DI, Clewley D, Young JL, Sissel CD, Cook CE (2018). Leveraging healthcare utilization to explore outcomes from musculoskeletal disorders: methodology for defining relevant variables from a health services data repository. BMC Med Inform Decis Mak..

[47] U.S. Coast Guard (2018). Coast Guard Occupational Medicine Manual. Vol COMDTINST M6260.32.

[48] Fowles J, Dybing E (2003). Application of toxicological risk assessment principles to the chemical constituents of cigarette smoke. Tob Control..

[49] Shane L III. ‘Deploy or get out’ policy may not have forced out any troops at all. Military. Times. 2019.

[50] O'Malley KJ, Cook KF, Price MD, Wildes KR, Hurdle JF, Ashton CM (2005). Measuring diagnoses: ICD code accuracy. Health Serv Res..

[51] O'Donnell FL, Stahlman S, Oetting AA (2018). Incidence rates of diagnoses of cardiovascular diseases and associated risk factors, active component, U.S. Armed Forces, 2007-2016. MSMR..

[52] Federal Register. Oil and Gas and Sulphur Operations on the Outer Continental Shelf—Oil and Gas Production Safety Systems—Revisions Bureau of Safety and Environmental Enforcement, Department of the Interior; 2017.

