# Supplementary material for: Risk of longer-term neurological conditions in the Deepwater Horizon Oil Spill Coast Guard Cohort Study – Five years of follow-up
Source: Environ Health. 2023 Jan 25;22:12. doi: 10.1186/s12940-022-00941-0 (PMC9875433; doi:10.1186/s12940-022-00941-0)
Supplement: Supplementary file 1 — Additional file 1. [file 12940_2022_941_MOESM1_ESM.docx]

**Supplemental Table 1. A full listing of neurological conditions and corresponding ICD-9 codes evaluated in the Deepwater Horizon Oil Spill Coast Guard Cohort**

| **3-digit ICD-9 code(s)** | **4-digit ICD-9 code(s)** | **5-digit ICD-9 code** | **Condition Description** |
| --- | --- | --- | --- |
| 346 |  |  | Migraine |
|  | 346.0 |  | Migraine with aura |
|  | 346.1 |  | Migraine without aura |
|  | 346.2 |  | Variants of migraine* |
|  | 346.3 |  | Hemiplegic migraine* |
|  | 346.4 |  | Menstrual migraine* |
|  | 346.5 |  | Persistent migraine aura without cerebral infarction* |
|  | 346.7 |  | Chronic migraine without aura* |
|  | 346.8 |  | Other forms of migraine* |
|  | 346.9 |  | Migraine, unspecified |
|  | 346.1-346.3, 346.5, 346.7-346.9 |  | Migraine excluding menstrual migraine and persistent migraine aura with cerebral infarction |
|  | 784.0 |  | Headache |
|  |  | 307.81 | Tension headache* |
| 339 |  |  | Other headache syndromes |
| 339,346 | 784.0 |  | Headaches/migraines combined |
| 339 | 346.1-346.3, 346.5, 346.7-346.9, 784.0 |  | Headaches/migraines combined excluding menstrual migraine and persistent migraine aura with cerebral infarction |
|  | 458.0 |  | Orthostatic hypotension* |
|  |  | 780.93 | Memory loss |
|  | 799.5 |  | Signs and symptoms involving cognition |
|  |  | 799.51 | Attention or concentration deficit |
|  |  | 799.52 | Cognitive communication deficit* |
|  |  | 799.53 | Visuospatial deficit* |
|  |  | 799.54 | Psychomotor deficit* |
|  |  | 799.55 | Frontal lobe and executive function deficit* |
|  |  | 799.59 | Other signs and symptoms involving cognition* |
|  | 333.1 |  | Essential and other specified forms of tremor |
|  |  | 333.94 | Restless legs syndrome |
| 351 |  |  | Facial nerve disorders |
| 352 |  |  | Disorders of other cranial nerves* |
| 353 |  |  | Nerve root and plexus disorders |
| 354 |  |  | Mononeuritis of upper limb and mononeuritis multiplex |
|  | 354.0 |  | Carpal tunnel syndrome |
| 355 |  |  | Mononeuritis of lower limb |
| 357 |  |  | Inflammatory and toxic neuropathy* |
|  | 356.4, 356.8, 356.9 | 357.89 | Peripheral neuropathy |
| 368 |  |  | Visual disturbances |
|  | 368.1 |  | Subjective visual disturbances |
|  | 368.2 |  | Diplopia* |
| 389 |  |  | Hearing loss |
|  | 388.3 |  | Tinnitus |
|  | 780.2 |  | Syncope and collapse |
|  | 780.4 |  | Dizziness and giddiness |
|  | 781.2 |  | Abnormality of gait |
|  | 781.3 |  | Lack of coordination* |
|  | 782.0 |  | Disturbance of skin sensation |

*excluded from analyses because <10 cases per exposure group (responder or non-responder)

| Supplemental Table 2. Risk of neurological conditions comparing active duty DWH-CG Cohort responders to non-responders, 2010-2015; ICD-9 diagnostic codes in first or second position | | | | | |
| --- | --- | --- | --- | --- | --- |
|  | **Responder (N=5964)** | | **Non-responder (N=39260)** | |  |
| **Condition (ICD-9 code)** | **N** | **Person Years** | **N** | **Person Years** | **HR* (95% CI)** |
| Migraine (346) | 135 | 25751 | 1036 | 172853 | 0.95 (0.80-1.14) |
| Migraine with aura (346.0) | 27 | 26473 | 161 | 177276 | 1.17 (0.77-1.76) |
| Migraine without aura (346.1) | 21 | 26474 | 206 | 176950 | 0.73 (0.47-1.15) |
| Migraine, unspecified (346.9) | 112 | 25908 | 798 | 174082 | 1.04 (0.85-1.27) |
| Migraine excl. menstrual migraine and persistent migraine aura with cerebral infarction (346.1-346.3, 346.5, 346.7-346.9) | 128 | 25809 | 944 | 173223 | 1.00 (0.83-1.20) |
| Headache (784.0) | 199 | 25495 | 1716 | 169285 | **0.82 (0.70-0.95)** |
| Other headache syndromes (339) | 73 | 26344 | 486 | 176284 | 1.03 (0.80-1.32) |
| Headaches/migraines combined (339, 346, 784.0) | 316 | 24716 | 2539 | 164871 | 0.89 (0.79-1.00) |
| Headaches/migraines combined excl. menstrual migraine and persistent migraine aura with cerebral infarction (339, 346.1-346.3, 346.5, 346.7-346.9, 784.0) | 314 | 24747 | 2482 | 165124 | 0.91 (0.81-1.02) |
| Memory loss (780.93) | 17 | 26553 | 125 | 177500 | 0.87 (0.53-1.45) |
| Signs and symptoms involving cognition (799.5) | 9 | 26567 | 55 | 177757 | 1.02 (0.50-2.06) |
| Attention or concentration deficit (799.51) | <9 | 26573 | 43 | 177770 | - |
| Essential and other specified forms of tremor (333.1) | <9 | 26569 | 45 | 177662 | - |
| Restless legs syndrome (333.94) | 21 | 26538 | 132 | 177425 | 1.08 (0.68-1.72) |
| Facial nerve disorders (351) | 15 | 26511 | 73 | 177499 | 1.42 (0.81-2.49) |
| Nerve root and plexus disorders (353) | 19 | 26515 | 133 | 177352 | 0.95 (0.58-1.53) |
| Mononeuritis of upper limb and mononeuritis multiplex (354) | 107 | 26194 | 746 | 175246 | 0.96 (0.79-1.18) |
| Carpal tunnel syndrome (354.0) | 75 | 26332 | 479 | 176217 | 1.06 (0.83-1.36) |
| Mononeuritis of lower limb (355) | 64 | 26423 | 392 | 176600 | 1.06 (0.82-1.39) |
| Peripheral neuropathy (356.4, 356.8, 356.9, 357.89) | 15 | 26527 | 103 | 177484 | 0.94 (0.55-1.62) |
| Visual disturbances (368) | 61 | 26331 | 430 | 176081 | 0.94 (0.72-1.24) |
| Subjective visual disturbances (368.1) | 13 | 26536 | 74 | 177537 | 1.18 (0.65-2.14) |
| Hearing loss (389) | 251 | 25629 | 1456 | 172530 | 1.12 (0.98-1.29) |
| Tinnitus (388.3) | 106 | 26292 | 718 | 175741 | 0.95 (0.77-1.17) |
| Syncope and collapse (780.2) | 55 | 26318 | 486 | 175530 | 0.78 (0.59-1.04) |
| Dizziness and giddiness (780.4) | 125 | 26034 | 821 | 174361 | 1.04 (0.86-1.25) |
| Abnormality of gait (781.2) | 38 | 26449 | 197 | 177034 | 1.27 (0.90-1.81) |
| Disturbance of skin sensation (782.0) | 121 | 26098 | 928 | 174210 | 0.87 (0.72-1.06) |

*Models adjusted for age, sex, and race

Bold indicative of statistical significance

| Supplemental Table 3. Risk of neurological conditions comparing active duty DWH-CG Cohort responders to non-responders after exclusion of 1,411 Occupational Medical Surveillance and Evaluation Program (OMSEP) enrollees; 2010-2015 | | | | | |
| --- | --- | --- | --- | --- | --- |
|  | **Responder (N=5722)** | | **Non-responder (N=38091)** | |  |
| **Condition (ICD-9 code)** | **N** | **Person Years** | **N** | **Person Years** | **HR* (95% CI)** |
| Migraine (346) | 149 | 24433 | 1149 | 166162 | 0.96 (0.81-1.14) |
| Migraine with aura (346.0) | 30 | 25245 | 176 | 170941 | 1.19 (0.80-1.75) |
| Migraine without aura (346.1) | 21 | 25248 | 228 | 170577 | 0.65 (0.42-1.02) |
| Migraine, unspecified (346.9) | 125 | 24613 | 911 | 167381 | 1.03 (0.85-1.24) |
| Migraine excl. menstrual migraine and persistent migraine aura with cerebral infarction (346.1-346.3, 346.5, 346.7-346.9) | 138 | 24508 | 1068 | 166515 | 0.96 (0.80-1.15) |
| Headache (784.0) | 235 | 24089 | 1990 | 161852 | **0.84 (0.73-0.96)** |
| Other headache syndromes (339) | 79 | 25104 | 545 | 169822 | 1.00 (0.79-1.27) |
| Headaches/migraines combined (339, 346, 784.0) | 354 | 23247 | 2840 | 157249 | 0.90 (0.81-1.01) |
| Headaches/migraines combined excl. menstrual migraine and persistent migraine aura with cerebral infarction (339, 346.1-346.3, 346.5, 346.7-346.9, 784.0) | 349 | 23302 | 2788 | 157518 | 0.91 (0.81-1.02) |
| Memory loss (780.93) | 19 | 25329 | 153 | 171132 | 0.80 (0.50-1.30) |
| Signs and symptoms involving cognition (799.5) | 13 | 25347 | 64 | 171445 | 1.27 (0.70-2.30) |
| Attention or concentration deficit (799.51) | 10 | 25353 | 51 | 171461 | 1.22 (0.62-2.41) |
| Essential and other specified forms of tremor (333.1) | 9 | 25349 | 56 | 171328 | 1.08 (0.53-2.19) |
| Restless legs syndrome (333.94) | 26 | 25285 | 194 | 170980 | 0.89 (0.59-1.35) |
| Facial nerve disorders (351) | 15 | 25294 | 80 | 171186 | 1.30 (0.74-2.26) |
| Nerve root and plexus disorders (353) | 23 | 25276 | 154 | 170974 | 1.00 (0.64-1.55) |
| Mononeuritis of upper limb and mononeuritis multiplex (354) | 118 | 24927 | 789 | 168769 | 1.01 (0.83-1.23) |
| Carpal tunnel syndrome (354.0) | 79 | 25094 | 505 | 169802 | 1.07 (0.84-1.36) |
| Mononeuritis of lower limb (355) | 72 | 25186 | 455 | 170103 | 1.04 (0.81-1.34) |
| Peripheral neuropathy (356.4, 356.8, 356.9, 357.89) | 16 | 25305 | 142 | 171083 | 0.75 (0.45-1.26) |
| Visual disturbances (368) | 85 | 24997 | 614 | 169101 | 0.94 (0.75-1.18) |
| Subjective visual disturbances (368.1) | 21 | 25295 | 106 | 171121 | 1.38 (0.86-2.22) |
| Hearing loss (389) | 276 | 24304 | 1656 | 165696 | 1.10 (0.96-1.25) |
| Tinnitus (388.3) | 125 | 24997 | 927 | 169021 | 0.87 (0.72-1.05) |
| Syncope and collapse (780.2) | 53 | 25100 | 520 | 169143 | **0.71 (0.54-0.94)** |
| Dizziness and giddiness (780.4) | 139 | 24759 | 969 | 167473 | 0.99 (0.83-1.18) |
| Abnormality of gait (781.2) | 65 | 25119 | 386 | 170032 | 1.11 (0.85-1.44) |
| Disturbance of skin sensation (782.0) | 143 | 24824 | 1161 | 167124 | **0.83 (0.70-0.99)** |

*Models adjusted for age, sex, and race

Bold indicative of statistical significance

| **Supplemental Table 4. Risk of neurological conditions among active duty DWH-CG Cohort responders reporting ever vs. never exposure to crude oil inhalation, 2010-2015, ICD-9 diagnostic codes in first or second position** | | | | | |
| --- | --- | --- | --- | --- | --- |
|  | **Oil inhalation ever (N=1068)** | | **Oil inhalation never (N=2424)** | |  |
| **Condition (ICD-9 code)** | **N** | **Person Years** | **N** | **Person Years** | **HR* (95% CI)** |
| Migraine (346) | 33 | 4733 | 54 | 10475 | 1.51 (0.97-2.35) |
| Migraine with aura (346.0) | 10 | 4876 | 13 | 10,790 | 2.16 (0.93-5.04) |
| Migraine, unspecified (346.9) | 23 | 4779 | 50 | 10530 | 1.10 (0.66-1.81) |
| Migraine excl. menstrual migraine and persistent migraine aura with cerebral infarction (346.1-346.3, 346.5, 346.7-346.9) | 31 | 4746 | 51 | 10501 | 1.48 (0.94-2.33) |
| Headache (784.0) | 52 | 4662 | 84 | 10359 | 1.41 (0.99-2.00) |
| Other headache syndromes (339) | 18 | 4844 | 26 | 10741 | **1.88 (1.01-3.52)** |
| Headaches/migraines combined (339, 346, 784.0) | 79 | 4502 | 133 | 10019 | **1.43 (1.07-1.90)** |
| Headaches/migraines combined excl. menstrual migraine and persistent migraine aura with cerebral infarction (339, 346.1-346.3, 346.5, 346.7-346.9, 784.0) | 78 | 4511 | 133 | 10032 | **1.40 (1.05-1.86)** |
| Mononeuritis of upper limb and mononeuritis multiplex (354) | 22 | 4818 | 38 | 10698 | 1.59 (0.92-2.74) |
| Carpal tunnel syndrome (354.0) | 14 | 4854 | 27 | 10737 | 1.39 (0.71-2.71) |
| Mononeuritis of lower limb (355) | 15 | 4871 | 24 | 10773 | 1.47 (0.76-2.83) |
| Visual disturbances (368) | 12 | 4829 | 28 | 10729 | 0.99 (0.50-1.97) |
| Hearing loss (389) | 46 | 4707 | 108 | 10438 | 1.06 (0.74-1.51) |
| Tinnitus (388.3) | 27 | 4854 | 43 | 10712 | 1.62 (0.99-2.67)** |
| Syncope and collapse (780.2) | 13 | 4858 | 28 | 10707 | 1.16 (0.59-2.28)** |
| Dizziness and giddiness (780.4) | 17 | 4837 | 57 | 10572 | 0.71 (0.41-1.23) |
| Abnormality of gait (781.2) | 10 | 4879 | 13 | 10782 | 1.65 (0.72-3.80) |
| Disturbance of skin sensation (782.0) | 25 | 4819 | 49 | 10629 | 1.19 (0.73-1.94) |

*Models adjusted for age, sex, race, and smoking;

** Because of the proportionality of hazards assumption violation for *tinnitus* and for *syncope and collapse* during 2010-2015 (Schoenfeld p<0.05), results from sub-period analyses were: 2010-2012: N_oil inhal ever_=7, N_oil inhal never_=19, HR=0.94, 95% CI: 0.39-2.30 and 2013-2015: N_oil inhal ever_ =20, N_oil inhal never_ =24, HR 2.15, 95% CI: 1.17-3.96 and 2010-2012: N_oil inhal ever_=3, N_oil inhal never_=16, HR=0.44, 95% CI: 0.13-1.52 and 2013-2015: N_oil inhal ever_ =10, N_oil inhal never_ =12, HR 2.35, 95% CI: 0.98-5.64, respectively.

Bold indicative of statistical significance

| **Supplemental Table 5. Risk of neurological conditions among active duty DWH-CG Cohort responders reporting ever vs. never exposure to crude oil inhalation after exclusion of 152 Occupational Medical Surveillance and Evaluation Program (OMSEP) enrollees; 2010-2015** | | | | | |
| --- | --- | --- | --- | --- | --- |
|  | **Oil inhalation ever (N=1012)** | | **Oil inhalation never (N=2328)** | |  |
| **Condition (ICD-9 code)** | **N** | **Person Years** | **N** | **Person Years** | **HR* (95% CI)** |
| Migraine (346) | 36 | 4430 | 62 | 9951 | 1.47 (0.97-2.24) |
| Migraine with aura (346.0) | 11 | 4588 | 14 | 10303 | 2.23 (0.99-5.03) |
| Migraine, unspecified (346.9) | 25 | 4481 | 57 | 10014 | 1.10 (0.68-1.77) |
| Migraine excl. menstrual migraine and persistent migraine aura with cerebral infarction (346.1-346.3, 346.5, 346.7-346.9) | 33 | 4444 | 57 | 9979 | 1.44 (0.93-2.23) |
| Headache (784.0) | 57 | 4354 | 97 | 9804 | **1.43 (1.02-2.00)** |
| Other headache syndromes (339) | 19 | 4553 | 30 | 10247 | 1.68 (0.93-3.05) |
| Headaches/migraines combined (339, 346, 784.0) | 84 | 4185 | 151 | 9457 | **1.41 (1.07-1.85)** |
| Headaches/migraines combined excl. menstrual migraine and persistent migraine aura with cerebral infarction (339, 346.1-346.3, 346.5, 346.7-346.9, 784.0) | 82 | 4197 | 150 | 9473 | **1.38 (1.05-1.82)** |
| Mononeuritis of upper limb and mononeuritis multiplex (354) | 27 | 4519 | 40 | 10193 | **1.84 (1.11-3.05)** |
| Carpal tunnel syndrome (354.0) | 16 | 4565 | 27 | 10243 | 1.59 (0.84-3.03) |
| Mononeuritis of lower limb (355) | 14 | 4589 | 30 | 10277 | 1.14 (0.60-2.18) |
| Visual disturbances (368) | 17 | 4534 | 37 | 10195 | 1.07 (0.60-1.93) |
| Hearing loss (389) | 49 | 4410 | 115 | 9933 | 1.08 (0.76-1.52) |
| Tinnitus (388.3) | 35 | 4546 | 47 | 10200 | **1.97 (1.25-3.10)** |
| Syncope and collapse (780.2) | 12 | 4578 | 28 | 10223 | 1.07 (0.54-2.13)** |
| Dizziness and giddiness (780.4) | 18 | 4546 | 66 | 10062 | 0.68 (0.40-1.16) |
| Abnormality of gait (781.2) | 13 | 4574 | 24 | 10253 | 1.19 (0.60-2.35) |
| Disturbance of skin sensation (782.0) | 28 | 4527 | 61 | 10114 | 1.03 (0.65-1.62) |

*Models adjusted for age, sex, race, and smoking;

**Because of the proportionality of hazards assumption violation for *syncope and collapse* during 2010-2015 (Schoenfeld p<0.05), results from sub-period analyses were: 2010-2012: N_oil inhal ever_=3, N_oil inhal never_=16, HR=0.43, 95% CI: 0.12-1.49 and 2013-2015: N_oil inhal ever_ =9, N_oil inhal never_ =12, HR 2.17, 95% CI: 0.88-5.34.

Bold indicative of statistical significance

| Supplemental Table 6. Risk of neurological conditions among never-smoking active duty DWH-CG Cohort responders reporting ever vs. never exposure to crude oil inhalation; 2010-2015 | | | | | |
| --- | --- | --- | --- | --- | --- |
|  | **Oil inhalation ever (N=555)** | | **Oil inhalation never (N=1333)** | |  |
| **Condition (ICD-9 code)** | **N** | **Person Years** | **N** | **Person Years** | **HR* (95% CI)** |
| Migraine (346) | 20 | 2513 | 38 | 5780 | 1.37 (0.79-2.37) |
| Migraine with aura (346.0) | 9 | 2580 | 9 | 5978 | **2.71 (1.06-6.91)** |
| Migraine, unspecified (346.9) | 12 | 2542 | 34 | 5822 | 0.92 (0.47-1.79) |
| Migraine excl. menstrual migraine and persistent migraine aura with cerebral infarction (346.1-346.3, 346.5, 346.7-346.9) | 18 | 2520 | 34 | 5801 | 1.37 (0.77-2.44) |
| Headache (784.0) | 28 | 2458 | 51 | 5707 | 1.36 (0.85-2.17) |
| Other headache syndromes (339) | 9 | 2570 | 13 | 5971 | 1.74 (0.74-4.12) |
| Headaches/migraines combined (339, 346, 784.0) | 46 | 2380 | 88 | 5525 | 1.35 (0.94-1.94) |
| Headaches/migraines combined excl. menstrual migraine and persistent migraine aura with cerebral infarction (339, 346.1-346.3, 346.5, 346.7-346.9, 784.0) | 45 | 2385 | 87 | 5535 | 1.35 (0.93-1.94) |
| Mononeuritis of upper limb and mononeuritis multiplex (354) | 14 | 2548 | 20 | 5951 | **2.08 (1.04-4.18)** |
| Carpal tunnel syndrome (354.0) | <9 | 2574 | 12 | 5975 | - |
| Mononeuritis of lower limb (355) | <9 | 2581 | 19 | 5960 | - |
| Visual disturbances (368) | 11 | 2549 | 19 | 5932 | 1.42 (0.67-3.01) |
| Hearing loss (389) | 27 | 2476 | 63 | 5785 | 1.19 (0.75-1.87) |
| Tinnitus (388.3) | 21 | 2558 | 20 | 5958 | 2.73 (1.47-5.08)** |
| Syncope and collapse (780.2) | <9 | 2574 | 19 | 5911 | - |
| Dizziness and giddiness (780.4) | 11 | 2556 | 40 | 5827 | 0.69 (0.35-1.36) |
| Abnormality of gait (781.2) | <9 | 2572 | 14 | 5962 | - |
| Disturbance of skin sensation (782.0) | 11 | 2560 | 32 | 5887 | 0.91 (0.46-1.82) |

*Models adjusted for age, sex, and race;

**Because of the proportionality of hazards assumption violation for *tinnitus* during 2010-2015 (Schoenfeld p<0.05), results from sub-period analyses were: 2010-2012: N_oil inhal ever_=5, N_oil inhal never_=8, HR=1.78, 95% CI: 0.57-5.54 and 2013-2015: N_oil inhal ever_ =16, N_oil inhal never_ =12, HR 3.37, 95% CI: 1.58-7.18.

Bold indicative of statistical significance

| **Supplemental Table 7. Risk of neurological conditions among active duty DWH-CG Cohort responders with Survey 2 data reporting different levels of crude oil inhalation exposure (N=3102), 2010-2015** | | | | |
| --- | --- | --- | --- | --- |
| **Condition (ICD-9 code)** | **N** | **Person Years** | **HR* (95% CI)** | **p-trend** |
| **Headache (784.0)**, n=145 |  |  |  |  |
| Never | 60 | 6277 | 1.00 |  |
| Rarely | 31 | 2940 | 1.11 (0.72-1.71) |  |
| Sometimes | 29 | 2482 | 1.28 (0.82-2.01) |  |
| Most of the time | 16 | 1070 | 1.62 (0.93-2.82) |  |
| All of the time | 9 | 529 | 1.97 (0.97-4.00) | **0.02** |
| **Headaches/migraines combined (339, 346, 784.0),** n=224 |  |  |  |  |
| Never | 94 | 6085 | 1.00 |  |
| Rarely | 48 | 2843 | 1.13 (0.79-1.60) |  |
| Sometimes | 45 | 2386 | 1.35 (0.94-1.94) |  |
| Most of the time | 23 | 1026 | 1.57 (0.99-2.48) |  |
| All of the time | 14 | 508 | **2.05 (1.16-3.61)** | **0.003** |
| **Mononeuritis of upper limb and mononeuritis multiplex (354),** n=61 |  |  |  |  |
| Never | 23 | 6524 | 1.00 |  |
| Rarely | 15 | 3069 | 1.41 (0.73-2.70) |  |
| Sometimes | 11 | 2568 | 1.44 (0.69-2.98) |  |
| Most of the time | 7 | 1115 | 2.18 (0.93-5.13) |  |
| All of the time | 5 | 555 | **3.84 (1.43-10.31)** | **0.008** |
| **Hearing loss (389),** n=154 |  |  |  |  |
| Never | 73 | 6339 | 1.00 |  |
| Rarely | 36 | 2975 | 1.02 (0.68-1.52) |  |
| Sometimes | 31 | 2492 | 1.15 (0.75-1.76) |  |
| Most of the time | 10 | 1080 | 0.93 (0.48-1.81) |  |
| All of the time | 4 | 546 | 0.81 (0.29-2.22) | 0.99 |
| **Tinnitus (388.3),** n=74 |  |  |  |  |
| Never | 24 | 6526 | 1.00 |  |
| Rarely | 17 | 3081 | 1.48 (0.79-2.75) |  |
| Sometimes | 22 | 2578 | **2.52 (1.40-4.53)** |  |
| Most of the time | 7 | 1121 | 1.93 (0.83-4.51) |  |
| All of the time | 4 | 555 | 2.51 (0.86-7.32) | **0.004** |

*Models adjusted for age, sex, race, and smoking

**Bold** indicative of statistical significance

Proportionality of hazards assumption was not violated in any of the models

| **Supplemental Table 8. Risk of neurological conditions among active duty DWH-CG Cohort responders reporting ever vs. never exposure to crude oil via inhalation, direct skin contact, ingestion, or submersion, 2010-2015** | | | | | |
| --- | --- | --- | --- | --- | --- |
|  | **Oil ever**  **(N=1908)** | | **Oil never (N=1584)** | |  |
| **Condition (ICD-9 code)** | **N** | **Person Years** | **N** | **Person Years** | **HR* (95% CI)** |
| Migraine (346) | 59 | 8287 | 43 | 6850 | 1.26 (0.84-1.89) |
| Migraine with aura (346.0) | 15 | 8600 | 10 | 7060 | 1.57 (0.68-3.62) |
| Migraine, unspecified (346.9) | 49 | 8352 | 37 | 6900 | 1.21 (0.78-1.88) |
| Migraine excl. menstrual migraine and persistent migraine aura with cerebral infarction (346.1-346.3, 346.5, 346.7-346.9) | 57 | 8307 | 37 | 6872 | 1.39 (0.91-2.13) |
| Headache (784.0) | 98 | 8150 | 64 | 6725 | 1.38 (0.99-1.91)** |
| Other headache syndromes (339) | 33 | 8524 | 18 | 7037 | **1.86 (1.01-3.41)** |
| Headaches/migraines combined (339, 346, 784.0) | 147 | 7851 | 100 | 6499 | **1.36 (1.04-1.77)** |
| Headaches/migraines combined excl. menstrual migraine and persistent migraine aura with cerebral infarction  (339, 346.1-346.3, 346.5, 346.7-346.9, 784.0) | 147 | 7867 | 97 | 6511 | **1.41 (1.08-1.84)** |
| Mononeuritis of upper limb and mononeuritis multiplex (354) | 45 | 8479 | 24 | 7001 | **1.88 (1.11-3.18)** |
| Carpal tunnel syndrome (354.0) | 27 | 8547 | 17 | 7031 | 1.58 (0.83-3.02) |
| Mononeuritis of lower limb (355) | 25 | 8595 | 20 | 7036 | 1.05 (0.57-1.92) |
| Visual disturbances (368) | 33 | 8493 | 23 | 6995 | 1.30 (0.74-2.27) |
| Hearing loss (389) | 95 | 8241 | 80 | 6821 | 1.07 (0.78-1.46) |
| Tinnitus (388.3) | 55 | 8526 | 32 | 6979 | **1.65 (1.04-2.62)** |
| Syncope and collapse (780.2) | 25 | 8525 | 17 | 7031 | 1.39 (0.73-2.65) |
| Dizziness and giddiness (780.4) | 44 | 8488 | 44 | 6877 | 0.93 (0.60-1.43) |
| Abnormality of gait (781.2) | 22 | 8563 | 15 | 7033 | 1.13 (0.58-2.21) |
| Disturbance of skin sensation (782.0) | 52 | 8466 | 40 | 6935 | 1.02 (0.67-1.55) |

*Models adjusted for age, sex, race, and smoking;

**Because of the proportionality of hazards assumption violation for *headache* during 2010-2015 (Schoenfeld p<0.05), results from sub-period analyses were: 2010-2012: N_oil ever_=61, N_oil never_=35, HR=1.74, 95% CI: 1.12-2.70 and 2013-2015: N_oil ever_ =37, N_oil never_ =29, HR 0.99, 95% CI: 0.60-1.64.

Bold indicative of statistical significance
